# Report of Deaths in Buffalo for the Month of July, 1855

**Published:** 1855-09

**Authors:** J. Root

**Affiliations:** Health Physician


ART. X.—*Report of Deaths in Buffalo for the month*

| DISEASES. | Males. | Females. | Total. | AGE. Under 1 year. Male. | Female. | 1 to 2 years. Male. | Female. |
|---|---|---|---|---|---|---|---|
| Atrophia, | .... | 1 | 1 | .. | .. | .. | 1 |
| Apoplexy, | 1 | 1 | 2 | 1 | .. | .. | .. |
| Brain Disease, | 1 | .... | 1 | .. | .. | .. | .. |
| Burned, | 1 | .... | 1 | .. | .. | .. | .. |
| Consumption, | 6 | 9 | 15 | 1 | 1 | .. | .. |
| Convulsions, | 2 | 4 | 6 | 2 | 4 | .. | .. |
| Congestion of Brain, | 1 | .... | 1 | .. | .. | 1 | .. |
| Cholera, | .... | 1 | 1 | .. | .. | .. | .. |
| Croup, | 1 | .... | 1 | .. | .. | 1 | .. |
| Cholera Infantum, | 5 | 1 | 6 | 4 | 1 | 1 | .. |
| Child Bed, | .... | 1 | 1 | .. | .. | .. | .. |
| Cholera Morbus, | 1 | .... | 1 | .. | .. | .. | .. |
| Dropsy, | 1 | 2 | 3 | .. | .. | .. | .. |
| " of Brain, | 1 | 2 | 3 | 1 | 1 | .. | .. |
| Diarrhœa, | 8 | 3 | 11 | 5 | 3 | .. | .. |
| Delirium Tremens, | 1 | .... | 1 | .. | .. | .. | .. |
| Drowned, | 5 | .... | 5 | .. | .. | .. | .. |
| Debility, | 3 | 5 | 8 | 2 | 4 | .. | .. |
| Disease of Bowels, | 1 | .... | 1 | .. | .. | 1 | .. |
| Dental Hæmorrhage, | 1 | .... | 1 | .. | .. | .. | .. |
| Dissipation, | 2 | 1 | 3 | .. | .. | .. | .. |
| Dentition, | 2 | 5 | 7 | 2 | 4 | .. | 1 |
| Degeneration of Kidney, | 1 | .... | 1 | .. | .. | .. | .. |
| Dysentery, | 1 | .... | 1 | .. | .. | .. | .. |
| Epilepsy, | .... | 1 | 1 | .. | .. | .. | .. |
| Fever Typhoid, | 1 | 2 | 3 | .. | .. | .. | .. |
| " Scarlet, | 3 | 1 | 4 | .. | .. | 2 | .. |
| " Typhus, | 1 | .... | 1 | .. | .. | .. | .. |
| "Febris Nervosa," | 1 | .... | 1 | .. | .. | .. | .. |
| Gangrena Oris, | 1 | .... | 1 | .. | .. | .. | .. |
| Heart Disease, | .... | 1 | 1 | .. | .. | .. | .. |
| Hydrocephalus, | 3 | 1 | 4 | 3 | .. | .. | .. |
| Inflammation of Bowels, | 1 | .... | 1 | .. | .. | 1 | .. |
| Murdered, | 1 | .... | 1 | .. | .. | .. | .. |
| Marasmus, | 2 | 1 | 3 | 1 | 1 | 1 | .. |
| Measles, | 1 | .... | 1 | 1 | .. | .. | .. |
| Malignant Pustule, | .... | 1 | 1 | .. | .. | .. | .. |
| Old Age, | .... | 2 | 2 | .. | .. | .. | .. |
| Pneumonia, | .... | 1 | 1 | .. | 1 | .. | .. |
| Premature Birth, | .... | 1 | 1 | .. | 1 | .. | .. |
| Still-born, | 3 | 2 | 5 | 3 | 2 | .. | .. |
| Spinal Meningitis, | .... | 1 | 1 | .. | .. | .. | .. |
| Stricture of Colon, | 1 | .... | 1 | .. | .. | .. | .. |
| Sun Stroke, | 1 | .... | 1 | .. | .. | .. | .. |
| Softening of Spinal Marrow, | .... | 1 | 1 | .. | .. | .. | .. |
| Scrofula, | 1 | .... | 1 | .. | .. | .. | .. |
| Unknown, | 9 | 6 | 15 | 5 | 6 | 1 | .. |
| Ulceration of Bowels, | 1 | .... | 1 | .. | .. | .. | .. |
| Whooping Cough, | 4 | 3 | 7 | 1 | 2 | 3 | .. |
| | | | | .. | .. | .. | .. |
| | | | | .. | .. | .. | .. |
| Totals, | 81 | 61 | 142 | 32 | 31 | 12 | 2 |

NATIVITIES.—American, 70. German, 28. English, 11. Irish, 16. French, 2.

*of July*, 1855. By J. Root, M. D., Health Physician.

| AGE. 2—5 | | 5—10 | | 10—15 | | 15—20 | | 20—25 | | 25—30 | | 30—40 | | 40—50 | | 50—60 | | 60—70 | | 70—80 | | 80—90 | | 90—100 | | 100 and upward. | | Not given. | |
|---|---|---|---|---|---|---|---|---|---|---|---|---|---|---|---|---|---|---|---|---|---|---|---|---|---|---|---|---|---|
| Male. | Female. | Male. | Female. | Male. | Female. | Male. | Female. | Male. | Female. | Male. | Female. | Male. | Female. | Male. | Female. | Male. | Female. | Male. | Female. | Male. | Female. | Male. | Female. | Male. | Female. | Male. | Female. | Male. | Female. |
| | | | | | | | | | | | | | | | | | | | | | | | | | | | | | |
| | | | | | | | | | | | 1 | | | | | | | | | | | | | | | | | | |
| | | | | | | | | | | | | | | 1 | | | | | | | | | | | | | | | |
| 1 | | | | | | | | | | | | | | | | | | | | | | | | | | | | | |
| | | | 1 | | | 2 | 1 | 1 | 1 | | 1 | | 1 | | 1 | 1 | 2 | 1 | | | | | | | | | | | |
| | | | | | | | | | | | | | | | | | | | | | | | | | | | | | |
| | | | | | | | | | | | | | | | | | | | | | | | | | | | | | |
| | | | | | | | 1 | | | | | | | | | | | | | | | | | | | | | | |
| | | | | | | | | | | | | | | | | | | | | | | | | | | | | | |
| | | | | | | | | | | | | | | | | | | | | | | | | | | | | | |
| | | | | | | | | | | | | | 1 | | | | | | | | | | | | | | | | |
| 1 | | | | | | | | | | | | | | | | | | | | | | | | | | | | | |
| | | | | | | | | 1 | | | 1 | | | 2 | | | | | | | 1 | | | | | | | | |
| | 1 | | | | | | | | | | | | | | | | | | | | | | | | | | | | |
| 3 | | | | | | | | | | | | | | | | | | | | | | | | | | | | | |
| | | | | | | | | | | | | | | 1 | | 1 | 1 | | | | | | | | | | | | |
| | | | | | | 1 | | 1 | | 2 | | 1 | | | | | | | | | | | | | | | | | |
| | | | | | | | | | | | | | | | 1 | | | | | | | | | | | | | | |
| | | | | | | | | | | | | | | | | | | | | | | | | | | | | | |
| | | | | | | 1 | | | | | | | | | | | | | | | | | | | | | | | |
| | | | | | | | | | | | 1 | | | | | | | | | | | | | | | | | | |
| | | | | | | | | | | | | | | | | | | | | | | | | | | | | | |
| | | | | | | | | | | | | | 1 | | | 1 | | | | | | | | | | | | | |
| | | | | | | | | | | | | | | | | | 1 | | | | | | | | | | | | |
| | | | | | | | | | | | | | | | | | | | | | | | | | | | | | |
| | | | | | | | | 1 | 1 | | | | | | | | | | | | | | | | | | | | |
| | | 1 | 1 | | | | | | | | | | | | | | | | | | | | | | | | | | |
| | | | | | | | | 1 | | | | | | | | | | | | | | | | | | | | | |
| | | | | | | | | | | | | 1 | | | | | | | | | | | | | | | | | |
| 1 | | | | | | | | | | | | | | | | | | | | | | | | | | | | | |
| | | | | | | | | | | | | | | | 1 | | | | | | | | | | | | | | |
| | 1 | | | | | | | | | | | | | | | | | | | | | | | | | | | | |
| | | | | | | | | | | | | | | | | | | | | | | | | | | | | | |
| | | | | | | | | 1 | | | | | | | | | | | | | | | | | | | | | |
| | | | | | | | | | | | | | | | | | | | | | | | | | | | | | |
| | | | | | | | | | | | | | | | | | | | | | | | | | | | | | |
| | | | | | | | | | 1 | | | | | | | | | | | | | | | | | | | | |
| | | | | | | | | | | | | | | | | | | | | | | | | | | | | | |
| | | | | | | | | | | | | | | | | | | | | | | | | | | | | | |
| | | | | | | | | | | | | | | | | | | | | | | | | | | | | | |
| | | | | | | | | | | | | | | | | | | | | | | | | | | | | | |
| | 1 | | | | | | | | | | | | | | | | | | | | | | | | | | | | |
| | | 1 | | | | | | | | | | | | | | | | | | | | | | | | | | | |
| | | | | | | | | | | | | 1 | | | | | | | 1 | | 1 | | | | | | | | |
| | | 1 | | | | | | | | | | | | | 1 | | | | | | | | | | | | | | |
| | | | | | | | | | | | | 1 | | | | | | | | | | | | | | | | | |
| 1 | | | | | | | | 1 | | 1 | | | | | | | | | | | | | | | | | | | |
| | | | | | | | | | | 1 | | | | | | | | | | | | | | | | | | | |
| | 1 | | | | | | | | | | | | | | | | | 1 | | | | | | | | | | | |
| | | | | | | | | | | | | | | | | | | | | | | | | | | | | | |
| | | | | | | | | | | | | | | | | | | | | | | | | | | | | | |
| 7 | 4 | 1 | 2 | 0 | 0 | 4 | 2 | 7 | 3 | 4 | 4 | 4 | 3 | 4 | 4 | 3 | 4 | 2 | 1 | 0 | 2 | 0 | 0 | 0 | 0 | 0 | 0 | 0 | 0 |

Canadian, 2. Hollander, 1. Country not given, 21.—Total, 142.

